# Viral Infections in HSCT: Detection, Monitoring, Clinical Management, and Immunologic Implications

**DOI:** 10.3389/fimmu.2020.569381

**Published:** 2021-01-20

**Authors:** Claudio Annaloro, Fabio Serpenti, Giorgia Saporiti, Giulia Galassi, Francesca Cavallaro, Federica Grifoni, Maria Goldaniga, Luca Baldini, Francesco Onida

**Affiliations:** Hematology–BMT Center, Fondazione IRCCS Ca’ Granda Ospedale Maggiore Policlinico, University of Milan, Milano, Italy

**Keywords:** hematopoietic stem cell transplantation, viral infection, adoptive immunotherapy, immunologic recovery, vaccines, cytomegalovirus

## Abstract

In spite of an increasing array of investigations, the relationships between viral infections and allogeneic hematopoietic stem cell transplantation (HSCT) are still controversial, and almost exclusively regard DNA viruses. Viral infections *per se* account for a considerable risk of morbidity and mortality among HSCT recipients, and available antiviral agents have proven to be of limited effectiveness. Therefore, an optimal management of viral infection represents a key point in HSCT strategies. On the other hand, viruses bear the potential of shaping immunologic recovery after HSCT, possibly interfering with control of the underlying disease and graft-*versus*-host disease (GvHD), and eventually with HSCT outcome. Moreover, preliminary data are available about the possible role of some virome components as markers of immunologic recovery after HSCT. Lastly, HSCT may exert an immunotherapeutic effect against some viral infections, notably HIV and HTLV-1, and has been considered as an eradicating approach in these indications.

## General Introduction

Optimal management of viral infections is a primary goal in every HSCT strategy in order to limit virus-related morbidity and mortality. Moreover, since viruses bear the potential of shaping immunologic recovery after HSCT, they possibly interfere also with control of the underlying disease and graft-*versus*-host disease (GvHD) and eventually with HSCT outcome. Finally, HSCT may exert an immunotherapeutic effect against some viral infections.

## Management of Viral Infections After HSCT

Management of viral infections after HSCT includes different steps. Monitoring of viremia and of virus-specific immune recovery are the main tools to drive anti-viral interventions after HSCT. Possible prognostic factors may help optimizing both patient prophylaxis and treatment. Immunotherapy, either active or more commonly adoptive, may provide alternatives to the limited effectiveness of the pharmacological agents and to their toxicities. Because most of the available data derive from the experience with cytomegalovirus (CMV), the main general issues about the different approaches will be presented in the *CMV* section, whereas in the sections dealing with other single viruses, virus-specific items will be mainly considered.

### CMV

CMV represents the most common viral reactivation after HSCT and the most deeply investigated. Serologic CMV-positivity has a high prevalence worldwide (over 80%), but with a rather wide inter-nation variability, therefore making donor/recipient (D/R) serologic mismatch a frequent problem in the setting of unrelated donor (1). Being of recipient origin in the majority of cases (2), the frequency of CMV reactivation after HSCT ranges from 10% in CMV-negative recipients to up to 90% in CMV-positive recipients with CMV-negative donor (3).

Due to its outmost adverse prognosis, CMV reactivation is the target of prophylaxis or pre-emptive therapy aimed at preventing end-organ disease. Being based on drugs associated with considerable toxicity, prophylaxis therapy has been formerly somewhat unpopular; moreover, concern is raised as to whether the ever-wider use of these drugs may enhance the development of viral drug-resistance (4). Among new drugs, however, letermovir has recently shown a very good safety profile and excellent efficiency, therefore being currently indicated for prophylaxis of CMV infection in adult CMV seropositive recipients of an allogeneic HSCT.

#### Monitoring

With the above premises, early detection of CMV reactivation is a key point to avoid undue treatments. Monitoring of CMV reactivation is routinely performed after HSCT, with quantitative PCR being largely considered more reliable than p65 antigenemia in driving timely pre-emptive therapy. Nevertheless, some concern still exists as to when pre-emptive therapy should be started. More recently an RNA-detecting transcription-reverse transcription concerted reaction (TRC) has been explored as an alternative diagnostic tool, but with a possible advantage in detecting a resolved viral activation rather than in timely recognizing its beginning (5).

Monitoring of specific anti-CMV immune reconstitution may represent an additional tool for predicting CMV reactivation, possibly optimizing the use of anti-CMV drugs and driving the referral to adoptive immunotherapy (**Table 1**). In general, an inverse relationship between CMV-specific immune recovery and CMV viremia appearance, severity and relapse has been clearly demonstrated; on the other hand, patients spontaneously clearing viremia develop a CMV-specific T-cell recovery (19, 22).

**Table 1:** Summary of experiences in monitoring specific CMV recovery.

References	Technique	Target	N. of patients	D/R serology	Type of transplant	Follow up	CMV recovery	Best recovery	Note
**Borchers et al. (** **6****)**	Tetramers, 7 commercial kits	1 CD8/mcL	278	All pairings	All represented	100 days	198/278, 71%	D+/R+	HLA-linked variability
**Nesher et al. (****7****)**	Elispot IE1 and p65	Spot count 50/100 according to cell threshold	63	R+	No haploidentical	100 days	42/63	D+/R+	21/23 CMV reactivations among negatives
**Cwynarski et al. (****8****)**	Tetramers	1 CD8/mcL	24	All pairings	13 sibling 11 MUD	100 days	9/24	D+/R+	No 100 day recovery after MUD
**Gratama et al. (****9****)**	Tetramers	1 CD8/mcL	27	All pairings	Sibling and MUD partially T-depleted	1 year	15/27	CMV reactivation without disease	1/9 recovery R-, 1/4 CMV disease
**Hebart et al. (****10****)**	Tetramers and ELISPOT	1/mcL	23	No D-/R-	Heterogeneous	100 days	14/19 CD8 , 11/18 CD4	NA	Possible dysfunction of CD8
**Mohty et al. (****11****)**	ELISPOT and tetramers	1 /mcL	54 ELISPOT 16/54 tetramers	All pairings	RIC, sibling	1 year	46/54	D+	
**Ohnishi et al. (****12****)**	ELISPOT and tetramers	1 /mcL	37	32D+/R+	Heterogeneous	250 days	All D+/R+	D+	ELISPOT earlier than tetramers, RIC earlier
**Lilleri et al. (****13** **)**	ELISPOT	3 CD8/mcL, 1 CD4/mcL	45	All pairings	MAC	1 year	63-98% according to D/R pairing	D+/R+	
**Gratama (****14** **)**	TEtramers	7 CD8/mcL	83	R+	No haploidentical	1 year	18% recovery at +65	D+/R+	+65 recovery predcitive of CMV disease
**Tormo et al. (****15****)**	ELISPOT	1 CD8/mcL, 1.2 CD4/mcL	133	All pairings	Heterogeneous	1 year	89.1% evaluable	D+	Delayed recovery after T-cell depletion and UCB
**Borchers (****16** **)**	Tetramers	10 CD8/mcL	134	All pairings	Heterogeneous	2 years	79 D+/R+ 58 D-/R+ 43 D+/R- at day 100	D+/R+	
**Lilleri et al. (****17****)**	Elispot	3 CD8/mcL, 1 CD4/mcL	131 pediatric or young	All pairings	Heterogeneous	1 year	76 R+(90% evaluable) 8 R- (21%)	D+/R+	No further CMV reactivation after CMV-specific recovery
**Tey et al. (****18****)**	Quantiferon	IFN-g >0.2 IU/mL	41	All pairings	Heterogeneous	1 year	31/41	No	
**Yong et al (****19****)**	Quantiferon	IFN-g >0.2 and >0.1 IU/mL	96	All pairings	No haploidentical	100 days	No response in 8 CMV disease	Spontaneous CMV control	Similar results with ELISPOT
**Krawczyk et al. (****20****)**	Quantiferon	IFN-g >0.2 IU/mL	48	All pairings	No haploidentical	1 year	5/9 D-/R+, 9/14 D+/R+	IFN >8.1 IU/mL protecting from viremia	Quantitative assessment of IFN response crucial for CMV control
**Paouri et al. (****21** **)**	Quantiferon	IFN-g >0.2 IU/mL	37	All pairings	Pediatric	1 year	15/37	Lower reactivation after specific CMV immune recovery	

D/R donor/recipient; MAC myeloablative conditioning; RIC reduced intensity conditioning; MUD matched unrelated donor.

Combining HLA-multimer-bound CMV-peptides and CMV-peptide-induced cytokines, notably gamma-interferon, has been proposed as a suitable method for recognizing CMV specific T-cell; peptides deriving from the CMV p65 and immediate-early 1 (IE-1) proteins have been regarded as the best promising viral markers to be detected (23). Other CMV proteins, as pUL97, have been more recently proposed as possible alternative or complementary diagnostic tools (24), while HLA multimers and cytokine release represent the favorite methods to retrieve virus-specific T-lymphocytes for adoptive therapy. A limitation of HLA-multimers is that they are HLA-restricted and recognized only by CD8 cells, being almost exclusively class I.

The main evolutions of the cytokine release-based CMV-specific T-cell recovery detection have been Quantiferon and Enzyme-Linked Immunospot Interferon-*γ*-Release Assay (ELISPOT), currently the most widely used technique, paralleling HLA-multimer-based ones (7, 25); HLA multimers and ELISPOT have also been combined and compared, leading to similar results (9). Conversely, Ohnishi et al. found that ELISPOT was more reliable than HLA multimers in early recognizing functionally active anti-CMV T-cells (10). Of note, HLA multimers allow cytotoxic T-lymphocytes (CTL) avidity to be evaluated, although this parameter recently failed to show predictive value in the setting of CMV immune recovery (26). More recently, streptamer technology has been a further development of HLA multimer-based methods (27).

Previous studies have suggested that anti-CMV immune reconstitution is a rather late event, earlier in case of donor seropositivity, hindered in T-cell depleted HSCT and independent of viral reactivation (23, 28). The late appearance of CMV specific T-cells, notably in case of donor (D)−/recipient (R)+ serology, has been widely confirmed (20). The independence of CMV immune reconstitution and CMV reactivation has not been confirmed in other experiences (25). On the other hand, failure to achieve a good expansion of CMV-specific T-lymphocytes after CMV reactivation is linked to the failure of spontaneous viremia clearance (29). The degree of HLA mismatch is a possible, additional factor negatively affecting CMV-specific immune recovery (30); on the other hand, HLA class I mismatch may be more likely associated with immunodominant genotypes in CMV antigen presentation (31).

Donor age has also been claimed to affect significantly the occurrence and the quality of CMV immune recovery (25), in accordance to investigation on healthy subjects that showed an evolution of CMV T-cell immunity with increasing age (32, 33). These issues may have a particular impact on patients receiving parent-derived haploidentical HSCT (34). Since T-lymphocytes from patients failing to clear CMV had a poorer cytokine release after challenge with CMV antigens, quality of CMV immune recovery is supposed to play an important role in controlling CMV reactivation (35). Moreover, a “memory cell type” response to lymphocyte proliferation assay predicts a better CMV-protection (36). The most recent and complex approach evaluated the CD8+ cytokine secretion profile in response to CMV antigens, with identification of a non-protective (NPS; IL-2-IFN-*γ*+TNF-α-MIP-1*β*+) and a protective signature (IL-2+IFN-*γ*+TNF-α+MIP-1*β*+), respectively linked to lack of control and control of CMV reactivation (37).

Although reduced intensity conditioning (RIC) has been early recognized as an additional transplant-related factor negatively interfering with CMV immune recovery (9, 38), D−/R+ combination is the main transplant related factor affecting CMV-specific immune reconstitution; indeed, this pairing does not simply delay the recovery of CMV-specific cells but also affects their pattern of cytokine release (39). A confounding factor may be sometimes the persistence or even the transient expansion of recipient derived anti-CMV T-cells, possibly interfering with establishing of full donor chimerism (40) following RIC conditioning (41).

In an attempt at quantifying T-cell recovery, a study investigating only CD8+ CTL proposed a threshold value of 1 × 10^7^ per liter (8). More recently, the threshold of 1 × 10^6^ anti-CMV CTL/liter two months after HSCT in the relatively more favorable D+/R+ setting was suggested as a protective level against viral reactivation (6). Overriding the matter of considering CD8 only or both CD8 and CD4, a threshold value of 3/µl for CD8 and of 1/µl for CD4 has been defined as protective after a prolonged follow-up in a series of young adults (17). Almost contemporarily, lower threshold values for CMV protection were also proposed, with counts of 1 and 1.2 cells/μl for CD8+ and CD4+, respectively (15).

#### Prognostic Factors

Serological donor–recipient mismatch, notably D−/R+ pairing, has been for long time recognized as the main *a priori* HSCT-related adverse prognostic factor for CMV reactivation and disease. Indeed, according to widely accepted recommendations, CMV serology is among the main donor selection criteria (3, 42). Other HSCT-related *a priori* risk factors include T-cell depletion, RIC conditioning and possibly unrelated cord blood (UCB) and haploidentical donor transplant. In the setting of RIC conditioning, the risk of CMV reactivation is delayed rather than increased, due to a delay of donor-type CMV-specific recovery and persistence of recipient CMV-specific lymphocytes (43, 44). In a multivariable analysis, D/R serostatus, GVHD and T-cell depletion resulted as independent predictors of CMV reactivation, enabling the authors to propose a risk score model (45).

In spite of considerable overlapping, GVHD and CMV-specific immune recovery are the best recognized *a posteriori* risk factors for CMV reactivation and severity. The aforementioned data show that CMV-specific immune recovery has a strong prognostic value even in the absence of GVHD and that GVHD is not the only shaping factor of CMV-specific immune recovery.

Furthermore, early NK response may have a favorable impact on the risk of CMV reactivation (36). There is a bidirectional relationship between NK recovery and CMV infection, since low NK level favors CMV reactivation and CMV reactivation shapes NK response, as specified in a subsequent section (46).

In spite of the high predictive value of these risk factors, considerable attention has been paid in order to identify additional, preferably patient- and/or donor-specific predictive elements.

A rather intuitive approach has been the correlation with particular HLA antigens. On this field, data are rather scant, with an increased risk for a negative CMV outcome only in HLA-DRB1*09 patients (47). More data are available on the presence of some class I MHC genotypes, known to be more efficient in presenting multiple CMV antigens; in different settings of HSCT, they seem to improve the outcome of HSCT possibly reducing the severity rather than the rate of CMV reactivation (31).

Donor KIR genotype has also been investigated, leading to the finding of a significantly lower risk of CMV reactivation if the donor had 5-6 KIR genes rather than 1–4 (48). However, this result should be taken cautiously, since only T-repleted, RIC HSCT from matched sibling donor were included in the analysis.

Gamma-delta lymphocytes bear the potential of exerting an antineoplastic and antiviral activity, possibly without eliciting GVHD, thus arousing the interest about a possible role in CMV control (49). On the other hand, CMV may shape gamma-delta recovery, as presented below. A recent meta-analysis showed a highly significant relationship between sustained post-HSCT gamma-delta recovery and protection against viral reactivations, mostly represented by CMV (50).

Attention has also been paid to single nucleotide polymorphisms (SNP) in key receptor or transcription factors. NOD-2/CARD 15 is an innate-immunity receptor, recognizing muramyl dipeptide, therefore mainly involved in antibacterial reactivity (51). Nevertheless, NOD2 polymorphism has been linked to the risk of developing other diseases, such as Crohn disease, and even to the outcome of HSCT (52). NOD2 is expressed on the surface of multiple cell lines, including monocytes, dendritic cells and NK cells. On a large unselected series, SNP8 mutation in either donor or recipient was linked to an increased risk of herpetic virus reactivation (51).

Forkhead box P3 (Foxp3) is a transcription factor that regulates T-reg development. In the limited setting of pediatric AML, HSCT recipients with donor-derived rs3761548 mutation showed a significantly increased risk of CMV reactivation (53).

Interleukin-7 (IL-7) is a hematopoietic cytokine essential for *de novo* T cell development in the thymus and homeostatic peripheral expansion of T cells signaling through the heterodimer IL-7 receptor (IL-7R). The IL-7R*α*-chain is a high affinity component expressed on naïve and memory T cells and downregulated in effector T-cells, which is also involved in TH2 differentiation and T-reg induction. Analysis of donor IL-7R*α* polymorphism showed that donor-derived homozygous SNP rs6897932 was significantly linked to CMV reactivation (54).

Rather surprisingly, a direct relationship has been described between CMV reactivation and early achievement of full donor chimerism in myeloablative HSCT recipient, irrespective of the CMV serostatus (55). This finding may be related to the aforementioned short-term CMV protective role of recipient-derived surviving T-cells (40).

#### Adoptive Immunotherapy

As previously stated, therapeutic failure is a common event in the treatment of CMV reactivation. In these cases, adoptive immunotherapy with original or third-party donor T-lymphocytes is generally regarded as the mainstay of treatment. General issues in manufacturing and infusing CMV-virus specific T-lymphocytes (VST) are common to the other virus infections to be presented below; on the other hand, most of the paper dealing with adoptive anti-viral immunotherapy is focused on CMV. Therefore, the general questions about adoptive immunotherapy will be described in this section and the analysis of the experiences in CMV treatment will be limited to the most relevant reports (**Table 2**).

**Table 2:** Adoptive immunotherapy as prophylaxis and treatment of CMV reactivation after HSCT.

References	Study	N. of patients	Type of transplant	Lymphocyte donor	Time to manufacturing	Method	Design	N. of cells	Result	GVHD
**Micklethwaite et al. (****56****)**	Phase I	9	Heterogeneous, HLA-A2, matched HSCT	Original donor	21 days	Stimulation with dendritic cells pulsed with p65 derived, HLA restricted peptide	Prophylaxis	2*10e7/sqm on day 28	2 subsequent selflimiting CMV reactivations	3 GVHD, 1 lethal
**Micklethwaite et al. (****57****)**	Phase I	12	Adults only, matched HSCT	Original donor	21 days	Stimulation with p65adenovector transduced dendritic cells	Prophylaxis	2*10e7/sqm On day 28	4 subsequent selflimiting CMV reactivations	4 grade II-III
**Peggs et al. (****58****)**	Phase I-II	18	Related donor adults	Original donor	24 hours	Overnight p65 challenge of unstimulated leukapheresis	11 pre-emptive, 7 prophylaxis	1*10e4/kg CD3+. About day 28	2/11 and 1/7 reactivations	3 grade II-III
**Uhlin et al. (****59****)**	Pilot	6	Heterogeneous	2 original donor, 4 third party haploidentical	2-4 hours	HLA restricted pentamers, within hours	Pre-emptive 2 toxicities, 4 refractory	>1*10e4/kg within 100 days	In 5/5 evaluable, long lasting viral clearance	Not reported
**Blyth et al. (****60****)**	Phase II	50	No haploidentical, CMV+ donor	Original donor	24 hours	HLA-restricted tetramers	Prophylactic/pre-emptive	2*10e7/sqm	5 post-infusion reactivation, 1 CMV death, reduced need of anti-CMV drugs	12 grade II-IV
**Koehne et al. (****61****)**	Phase I	17	Matched T cell depleted	16/17 original donor	28 days	Culture with conditioned APC	12 refractory viremia, 5 CMV disease	>5*10e5/kg day 98-164	12/12 and 3/5 clearance of viremia	No “de novo” or flare
**Neuenhahn et al. (****30****)**	Phase I-IIa	16	Heterogeneous	8 original donor, 8 third party	24 hours	HLA-restricted streptamer	Drug refractory viremia, pre-emptive	1*10e4/kg CD3+.	62,5% clearance of viremia	No

The original hematopoietic stem cell (HSC) donor is the preferable source of CMV-VST. Unfortunately, the original donor may be seronegative. Moreover, an unrelated donor may be variously unavailable for a further donation. In these cases, it is common to derive cell products from a third-party donor, generally a haploidentical related donor. Third party lymphocytes have been retrieved up to 60–90 days from infusion (62). A possible alternative method is the “off the shelf” approach, where VST, general with multi-virus specificity, are banked from unrelated third-party donors and delivered to suitable HLA-recipients. The multi-virus specificity reduces the costs of banking and offers an effective cell product in case of multiple viral infections (63).

The first approach to adoptive immunotherapy in CMV has been the delivering of polyclonal donor-derived T-cells, which were activated and expanded *ex-vivo* through the exposition to viral antigens. A major limit is the 4–8 weeks-time required to manufacture the cell product, which makes it unsuitable as a therapeutic strategy; therefore, a prophylactic or pre-emptive design was frequently preferred, where clearance of viremia and CMV immune recovery were the targets (56, 64). Although GVHD was not a negligible issue on the field of toxicity, the risk of such complication seems to be significantly lower with the refinement in antigen selection and a two logs (from 10^7^ to 10^5^/kg) decrease in the dose of infused cells (61).

CMV has also been included among the specificities of multi-viral VST, realized by *ex-vivo* challenge through multi-antigens peptide mixes (65). *Ex-vivo* expansion is the only suitable approach when even seropositive donors have a low rate of circulating specific T-cells, as it may happen with ADV. Conversely, CMV-specific T-lymphocytes account for at least 1% of the total T-lymphocytes in seropositive healthy subjects, apparently increasing with age (32, 33). Therefore, the short-term recovery of a sufficient amount of CMV-reactive cells seems to be a rationale purpose.

As for lymphocyte selection, two main methods are favored to retrieve CMV-specific T cells: HLA-multimer selection and gamma-interferon capture magnetic immunoselection.

The multimer selection is based on the recognition by CD8+ cells of class I HLA-multimers-bound CMV-derived and HLA-specific peptides. Tetramers are the reference HLA-multimers (66). Unfortunately, their steric configuration does not allow cell binding to each of the four sites (27). To overcome this limitation, advances in HLA multimerization have led to pentamers and octamers. Pentamers have been proposed as the best steric configuration because all five HLA-peptide complexes are available for T-cell binding (67). Conversely, octamers binding may induce T-cell apoptosis (68). Moreover, the avidity of conventional multimer binding causes a persistent antigen-T-cell interaction, possibly leading to functional impairment of the selected T-cells (27).

Streptamers are an evolution of the technique, having the advantage of a reversible binding to the target and allowing an easy detachment of the selected T cells (69). They are peptide-loaded Strep-tagged HLA monomers, binding CD8 at low affinity. Strep-Tactin multimerizes streptamers and increases T cell avidity. Finally, T-cells are displaced by the addition of biotin, binding Strep-Tactin with a higher affinity (69).

In a comparative study including tetramers, pentamers and steptamers, all of the methods proved to be reliable; nevertheless, tetramers gave the best results in terms of specificity, whereas streptamers allowed the achievement of a GMP-compliant product (66). As previously reminded, the best specificity of tetramers may be at the expense of quantitative yielding of CD8+ cells (27). However, a reduction in the number of required CD8+ cells for CMV treatment is reasonable in comparison to polyclonal CMV-stimulated cells, possibly about 1 × 10^4^/kg (59).

Rapid manufacturing of GMP-compliant cell products is the outstanding advantage of multimer selection, which was the chosen strategy in a recent phase I/II trial (30). The selection of only CD8+ cells is felt as a disadvantage, while the selection of CD4 through HLA class II multimers presents quite different problems and is still in a preliminary phase (70). On the other hand, the critical role of targeting multiple epitopes has been recently underscored (71), even though previous studies have shown its feasibility (72).

Gamma-IFN capture and immunomagnetic selection is the main alternative method. Short-term *ex-vivo* challenge with CMV antigens induces T-cells’ release of gamma-IFN; secreting cells undergo immunomagnetic selection after labeling with a double moAb conjugate, including a gamma-IFN and a CD45 directed moAb. Unlike multimer selection, this method allows both CD4 and CD8 to be collected, with CD4 generally accounting for the majority. The reported number of infused cells is variable, but a lower requirement is likely also in this setting, since 1–2 × 10^4^/kg CD3 cells have been repeatedly reported (58, 73).

As multimer selection, gamma-capture allows the rapid production of an active cell product, though requiring a short (overnight) *ex-vivo* expansion phase. The yield is lower in comparison to multimers entailing the processing of larger blood volumes, thus raising some concern as to the feasibility in case of unrelated donors. A definite advantage of gamma capture is the possibility of delivering both CD4 and CD8 cells, recognizing multiple viral epitopes. On the other hand, the method selects only gamma-IFN producing cells, whereas the diversification in cytokine profile could exert a role in achieving an effective anti-CMV response (39). Moreover, gamma-IFN alone, does not help discriminating between protective and non-protective cytokine release profiles (37).

In summary, few phase II clinical trials are available, with study design rather heterogeneous, ranging from prophylaxis (60) to pre-emptive therapy (30, 61). Low risk of GVHD, viremia clearance rates ranging from 60 to 100%, some response also in patients with CMV disease and low CMV-specific mortality are the outstanding findings (74, 75).

The experience with anti-CMV VST suggests some additional advantage of this approach. Effective adoptive immunotherapy proved to be linked to overall T-cell recovery (76) and to an improvement in CMV-related inhibition of hematopoiesis (77).

Conversely, the whole matter of adoptive immunotherapy suffers the general drawback of excluding patients with active GVHD, hindering the access to many severe cases. The reason obviously relies on the direct lympho-toxicity of corticosteroids and in the inhibiting activity of immunosuppressive agents. To solve this primary problem, investigations are ongoing to make VST resistant to immunosuppressive agents. The likely most intuitive approach of engineering cells through *ex-vivo* manipulation has not been explored so far (75). Conversely, Menger et al., in streptamer-selected CMV-specific CD8+ cells were able to disrupt the glucocorticoid receptor gene using electroporation of transcription activator-like effector nuclease messenger RNA (78). More recently, Basan et al. were able to produce GMP-compliant NR3C1- multi-virus VST (79). Among the other immunosuppressive agents, resistance to calcineurin inhibitors is under study, but the available data regard only EBV (80).

Tapering of immunosuppressive therapy represents a further open issue possibly leading to an over-estimation of adoptive immunotherapy-related GVHD. Indeed, at times, immunosuppression had been reduced or discontinued before starting adoptive immunotherapy, to avoid treatment interfering and/or to favor the clearance of viremia. To avoid third party lymphocytes and the inherent issues, the introduction of a virus-specific TCR in T-cells of original HSC donors failing to mount spontaneously a virus-specific response has also been explored. However, although preliminary data have been reported even on the field of CMV (81), this approach seems more suitable for viruses where the issue of a failure of T-cell response is a more compelling problem.

An alternative strategy is the generation and expansion of VST starting from naïve cells of seronegative donors or UCB, overcoming the risk of unavailability of the original HSC donor and allowing VST to be available before transplantation. Hanley et al. were able to expand tri-virus (adenovirus, CMV and EBV) specific T-cells from UCB units recognizing multiple viral epitopes (82). Later, they also observed that high avidity anti-CMV T-cells generated from naïve cells of seronegative donors had different epitope-specificities than high avidity T cells from seropositive healthy subjects, but proved to be effective in clearing viremia (82). Moreover, healthy subjects had low-avidity anti-CMV T-cells, recognizing the same epitopes as high avidity ones generated from naïve cells. Overall, these findings underscore the evolution over time of CMV-specific T-cells immunoreactivity in healthy subjects (32, 33).

#### Vaccines

Among post-HSCT viral infections, CMV is the only one where alternative ways to adoptive cell immunotherapy have been explored. Unfortunately, the available data are derived from preliminary, phase I–II clinical trials, generally not followed by investigations on larger series.

The best studied strategy has been patient active immunization. A commercial CMV-vaccine containing plasmids encoding glycoprotein B and phosphoprotein 65 was delivered to HSCT recipients in a randomized phase 2 study, with rather equivocal results (83).

A more promising approach seemed the administration of dendritic cells (DCs), variously challenged with viral components. In a preliminary study, CMV pp65 messenger RNA-loaded autologous monocyte-derived DC was administered to a small group of healthy subjects and HSCT recipients. Three of four healthy subjects and one of two evaluable HSCT recipients developed a detectable increase in CMV-specific T cells (83).

Taking into account the role of donor derived DC in the post-HSCT immune reconstitution, Sundarasetty et al. transduced peripheral blood monocytes with an integrase-defective lentiviral vector, co-expressing GM-CSF, IFN-α and the CMV antigen pp65, attaining the production of GMP-compliant donor-derived DCs suitable for clinical use (84).

An alternative approach has been the delivery, as a vaccine, of a chimeric peptide composed of a CD8-T-cell epitope from CMV pp65 and a tetanus T-helper epitope (CMVPepVax). As a common limitation of such products, the vaccine was HLA-restricted and was administered only to HLA-A*0201 HSCT-recipients. In a randomized phase 1b trial, CMVPepVax proved to be safe and treated patients had a significant reduction in NRM (85).

In a phase 2 trial, the ASP0113 vaccine, containing two plasmids encoding CMV antigens, was delivered to ten HSCT recipients in order to enhance both humoral and cellular immunity. Although the treatment showed a favorable toxicity profile, the clinical activity was questionable (86).

In a phase I trial, a small series of HSCT recipients received a CMVpp65-derived peptide as a CMV-vaccine. Most of the patients had a significant increase in CMV-specific CD8+ T cells and/or V*δ*2negative *γδ* T cells, and a humoral response of neutralizing antibodies, suggesting a correlation between the immune response and virus clearance (87).

### EBV

The relevance of EBV reactivation after HSCT relies on its pathogenetic role in the development of post-transplant lymphoproliferative disease (PTLD), recognized as a self-standing main lymphoma group in the recent WHO classification (88). The limited efficacy of the available therapeutic resources makes management of EBV reactivation a crucial issue.

In the vast majority of cases, PTLD after HSCT shows B-cell phenotype and is of donor origin (89). Conversely, analysis of LMP-1 polymorphism shows that EBV strains causing PTLD are mostly of recipient origin, although transmission of donor-derived EBV has been reported even in the setting of cord transplantation (90).

Immunophenotypically, expression of EBNA 2 and 3 is a characteristic of PTLD (91). Proliferation of EBNA 3+ lymphoid cells does not occur in the absence of an immunologic impairment, thus linking immunosuppression with PTLD (92).

Multiple *a priori* risk factors for the development of PTLD have been recognized, most of them concerning the type of transplant. HLA-mismatch, RIC conditioning, D/R serological mismatch, acute GVHD, and pre-transplant splenectomy proved to be predictive of PTLD development (93). HLA-mismatch includes also UCB transplant (94) whereas any kind of T-cell depletion has been recognized as a likely additional risk factor (95). On the field of haploidentical HSCT and therefore of HLA mismatch, patients receiving post-HSCT CTX may build up a subset at lower risk of PTLD, possibly attributable to lysis of EBV-infected lymphocytes with relative sparing of memory cells (96).

Beyond the aforementioned GVHD, the use of mesenchymal cell has been claimed to be an additional *a posteriori* risk factor, although the question can be raised as to its independence from GVHD (97).

Gamma/delta lymphocytes, notably delta-2+ recovery, have aroused considerable interest as a possible major EBV controlling factor. In experimental models, delta-2+ lymphocytes proved to be cytotoxic against EBV infected cells (98); in another study, delta-2+ recovery seemed to exert a protective role from EBV reactivation (99). The same authors showed that mycophenolate-driven inhibition of delta-2+ gamma-delta lymphocytes could play a role in the pathogenesis of PTLD, at least in the haploidentical setting (100).

Little is known about D/R specific risk factors. Patient age over 50 years is commonly considered as an adverse risk factor (101). Moreover, attention has been driven to the possible lymphocyte senescence in case of parental donor, possibly leading to a reduced antiviral response (34). In a previous study, EBV reactivation was more common among HSCT recipients with gamma-interferon 3/3 genotype (102). With some resemblance to the matter in HIV patients, the same authors showed a reduced risk of EBV reactivation in HSCT recipients heterozygous for CCR5/delta 32, in comparison to wild type homozygosity (103).

#### Monitoring

Monitoring of EBV viremia has been routinely performed after HSCT since many years (104). Parallel monitoring of EBV-specific T-lymphocytes recovery is not routinely performed, although it could offer clues in order to better understand the risk of PTLD development and to optimize rituximab treatment. EBV-specific T-cell recovery was shown to occur earlier compared to CMV in case of D/R serologic mismatch (105). Using the HLA class I tetramer technique to disclose EBV-specific CD8, Clave et al. showed that patients with EBV reactivation having virus-specific CD8 recovery had spontaneous resolution of viremia without rituximab, suggesting that monitoring of immune recovery could drive the administration of rituximab more than the viremia itself (106). Using the alternative ELISPOT technique, disclosing both specific CD4 and CD8 cells, there was a striking difference in EBV-specific immune recovery between patients transplanted with myeloablative (MAC) and RIC, suggesting a negative impact of the latter on the risk of developing PTLD (107). As a general comment, lack of EBV-specific immune recovery can be proposed as *a posteriori* risk factor for PTLD.

#### Adoptive Immunotherapy

Failure to achieve a stable response to rituximab has led to the use of adoptive immunotherapy in PTLD (108). Unselected donor lymphocytes (DLI) have been the oldest choice to treat refractory PTLD. This treatment fails to control PTLD when an *in-vivo* expansion of EBV-specific lymphocytes does not occur (109).

The risks connected to the use of unselected DLI and its limited effectiveness lead to the development of strategies to deliver EBV-specific cells. In normal EBV-seropositive subjects EBV-specific T-cells account for more than 1% of total T-lymphocytes, making the retrieval of a sufficient number of cells easy both for *ex-vivo* stimulation and for the manufacturing of a short term cell product (70, 109). A primary warning derived from a report showing that routine *ex-vivo* cytokine-induced expansion with recipient EBV-infected lymphoblastoid cells led to a loss of EBV reactivity in EBV-seropositive healthy donors, whereas unstimulated T-cells maintained their activity (110). This finding has not received further support, and does not seem to correspond to the clinical results with stimulated VST (111).

The relative ease of obtaining EBV-specific cells and the expected decreased risk of GVHD has led to a broadening of VST spectrum of indication, including not only proved PTLD but also high-risk virus reactivation (**Table 3**).

**Table 3:** Adoptive immunotherapy for PTLD treatment.

References	Type of study	N. of patients	Type of transplant	Donor	Technique	Tim to manufacturing	Time and dose	Purpose	Outcome	GVHD
**Moosmann et al. (****112****)**	Unspecified	6	Hetrogeneous	Original donor	Stimulation with EBV Antigens	Overnight	0.4-9.7*10e4/kg, according to need wihin 100 days	Refractory PTLD, therapeutic	3/6 PTLD remissions	No
**Doubrovina et al. (****109****)**	Unspecified	30	Mostly HLA matched	Original donor	DLI		0.2-1*10e6/kg, clinical need	Biopsy proven PTLD, therapeutic	73% remissions	17%
**Doubrovina et al. (****109****)**	Unspecified	19	Mostly HLA-mismatched	Original donor 14 and third party 5	Culture with	28-35 days	1*10 e6/kg, clinical need	Biopsy proven PTLD, therapeutic	68% remissions	No
**Heslop et al. (****113****)**	Unspecified	101	Mostly HLA-matched BM	Original donor	Culture with virus activated lymphoblastoid cell	28 days	1*10e7/sqm	Prophylaxis in high risk patients	No PTLD	7/101 grade I-II recurrence
**Heslop et al. (****113****)**	Unspecified	13	Mostly HLA-matched BM		Culture with virus activated lymphoblastoid cell	28 days	1*10e7/sqm	Therapy of biopsy proven PTLD	11/13 remissions	1/13 low grade recurrence

The use of multi-specific antiviral T-cells requires longer time than EBV-specific cells and is possibly more suited for other types of viral infections (65, 114, 115). Third-party donor cells have also been explored (116). Results have been reported according to the “off-the-shelf” approach, with donor cells still detectable 12 weeks after a single infusion (63).

The high rate of EBV-specific T-cells in seropositive healthy subjects facilitated the short-term release of potentially effective cell products, although the available clinical data are still limited. Interferon-gamma surface capture with immunomagnetic separation, allowing the recovery of both CD4 and CD8 VST, has been the favorite technique to retrieve short-term, donor-derived, EBV-reactive T-cells (117). Unfortunately, response to cell therapy appears to be limited to patients with less clinically advanced PTLD, with durable PTLD control being related to early *in-vivo* expansion similar to DLI (117). The selection technique through virus peptide bound to HLA class I multimers has been developed also for the selection of EBV specific CD8+ lymphocytes, but clinical data are still awaited (118).

Most of the available data derive from VST obtained through *ex-vivo* stimulation of donor lymphocytes with EBV infected lymphoblastoid cells. The largest series report favorable outcomes both when VST was used as treatment, with 11/13 patients achieving a response and when used as a preventive measure, with none of 101 treated patients developing PTLD. No new acute GVHD was recorded, and only 5.7% of patients experience grades I–II GVHD relapse (113).

In the specific setting of EBV, preliminary data have been reported about the generation of resistance to calcineurin inhibitors in virus-specific CTL (80, 119).

A subsequent step has been the development of EBV-specific cytokine-induced killer (CIK) cells, with a patient affected by VST-refractory PTLD being the first reported case. Unstimulated original donor mononuclear peripheral cells were expanded in the presence of interferon-*γ*, anti-CD3, IL-2 and IL-15, and pulsed with a commercial “EBV-select” peptide pool. CD3+CD56− cells, mainly CD8+, accounted for 89% of the cells in the final product, with the remaining being represented by CD3+CD56+ cells. CD3− CD56+ NK were almost undetectable. In the treated patient, a single EBV-specific CIK cells infusion achieved complete and durable resolution of a multi-resistant PTLD, with specific CIK cells being detectable until 30 days after the infusion (120).

### Adenovirus

Adenovirus (ADV) infections are a relevant cause of morbidity and mortality in HSCT patients, with pediatric patients and patients receiving highly manipulated or mismatched HSCT being at a particularly elevated risk (121, 122).

Adenovirus requires post-transplant viremia monitoring. Monitoring of ADV-specific lymphocyte recovery has also been proposed for many years, at least in the setting of pediatric high-risk transplantation (122), with ELISPOT being the favorite method. The aim of this strategy is to identify in advance patients requiring treatment with donor-derived ADV-specific T-cells, although the real benefit of monitoring has also been questioned (123). Failure of developing ADV-specific T-cells has been reported as a rather common problem, frequently associated with the appearance of ADV viremia/infection (124).

#### Adoptive Immunotherapy

Delivery of allogeneic ADV specific T-lymphocytes seems to be a reasonable way of treatment in patients with severe and/or refractory ADV infection (**Table 4**). Some specific ADV-related problems have been identified. With rare exceptions, ADV-specific T-cells account for a small proportion of total lymphocytes even in reactive donors, therefore requiring *ex-vivo* expansion to achieve count suitable for clinical purposes (126). Moreover, failure to retrieve ADV-specific cell in up to 20% of donors (127) frequently forces to use third party cells, generally from haploidentical related donors. Finally, failure to achieve a sufficient *ex-vivo* cell expansion from donors with baseline ADV-reactivity is an uncommon but well-established additional problem (62). A further matter of concern is the imbalance favoring CD4 on CD8 T-cells (128) and the low representation of central memory cells in the expanded T-cell population (126).

**Table 4:** Treatment of HSCT with multispecific VST.

References	Study	N. of patients	Type of transplant	CTL Donor	Specificity	Technique	Time to manufacturing	N. of cells	Time of delivery	Design of the study	Results	GVHD
**Papadopoulou et al. (****65****)**	Not stated	11 (3 prophylaxis and 8 with up to four active virus infections)	Heterogeneous, mostly pediatric	Original HSC donor	AdV, EBV, CMV, BKV,HHV6	In vitro culture, challenge with multiple viral antigens	9-11 days	0.5-2*10e7/sqm	38-139 days after HSCT	Therapeutic in 8/11	94% responses	No
**Gerdemann et al. (****115****)**	Not stated	10	Heterogeneous	Original donor	CMV, EBV, ADV	In vitro culture	14-21 days	0.5-2*10e7/sqm	28-222 days after HSCT	Therapeutic	80% response	
**Ma et al. (****125****)**	Not stated	10	Heterogeneous	Original donor	CMV, EBV, VZV, ADV	In vitro culture	2-3 weeks	2*10e7/sqm	Median 63 days after HSCT	Prophylaxis	6/10 CMV reactivation	3/10 grade II-IV GVHD
**Tzannou et al. (****63****)**	Phase II	56 patients followed, 38 treated	Heterogeneous	Third party	CMV, EBV, HHV6, BKV, ADV	In vitro culture	9-11 days	2*10e7/sqm	1-3 infusions starting within 100 days	Therapeutic	92% response rate	2 de novo GVHD grade I

Apart from some attempts to generate ADV-reactive cells in the setting of multi-virus T-lymphocytes products (129), many attempts have been made in order to solve the abovementioned ADV-specific issues. Anticipating the delivery of virus-specific T-lymphocytes in a preventive approach is an option, with the consequent disadvantage of a larger number of patients treated and exposed at risk of developing GvHD (62). The CAR-T technique has also been proposed to generate T-lymphocytes with double anti-CD19 and antivirus specificity, to treat B-cell ALL patients at high risk of both relapse and of virus infection (111). To overcome the problem of the lack of baseline and *ex-vivo* ADV-reactivity, transfer of T-cell receptor has been successfully performed, both in alpha/beta and in gamma/delta T-lymphocytes (127). With regard to the duration of response, third party ADV specific cells have been detected two months after infusion (62), whereas the limited proportion of CD45RA-/CCR7+ central memory cells achievable may be due to a weak effectiveness of the commonly used IFN-*γ*-based immunomagnetic selection system, claiming for the development of alternative selection techniques (130). A possible alternative could be the generation and expansion of ADV-specific T-cells from naïve donor lymphocytes; preliminary data are available on UCB, but no further development has been so far reported (82).

With these premises, two phase I/II clinical trials have been conducted on HSCT recipients (75, 131). In the former, IFN-gamma immunomagnetic selected anti-ADV T-lymphocytes from HSCT donor or third party haploidentical donor were administered after short term *ex-vivo* expansion to patients with refractory ADV infection. Manufacture failure occurred for 3/14 patients. CD4+ cells accounted for the vast majority of infused cells and virus clearance was achieved in 10/11 treated patients, with anti-ADV activity being detectable up to 90 days. GVHD risk was acceptable (75). The second trial followed the approach of previously collecting HSCT-donor-derived lymphocytes, either from mobilized PBSC or from lymphocyte apheresis and administering ADV-specific lymphocytes as a pre-emptive treatment. Again, failure to retrieve a suitable number of ADV-specific cells was reported in a substantial proportion of cases. All of the eight patients receiving ADV-specific cells as pre-emptive therapy achieved viral clearance. Grade II GVHD occurred in 1/8 patients (131).

### HHV6

HHV6 reactivation occurs in up to 50% of patients undergoing HSCT, showing a quite heterogeneous clinical counterpart ranging from asymptomatic carrier to severe end organ disease, with pediatric and UCB recipients being at the highest risk of severe complications (132, 133). The available therapeutic resources are far from being optimal, thus requiring development of further strategies.

Monitoring of viremia is commonly performed, especially in pediatric and in high risk HSCT (132–134). Conversely, monitoring HHV6 specific immune recovery has not been widely implemented. In a recent paper, a CD8 response against multiple HHV6 antigens was demonstrated by means of *ex vivo* HLA-multimer staining techniques, even though with the weakness of being HLA restricted and able to identify only CD8 T-cells (135).

Little is known about possible specific donor/recipients related factors affecting the HHV6 viral reactivation risk. A significant relationship between HHV6 CNS involvement and HLA class I genotype has been described, notably HLA-B*40:06 (136).

With regard to adoptive immunotherapy, predominantly multi-virus products have been used; HHV6-specific T-lymphocytes account for a scant minority of total lymphocytes in normal subjects, making *ex-vivo* expansion mandatory in order to achieve a sufficient number of T-lymphocytes. Gerdemann et al. reported the “short term” production of multi-specific anti-virus CD4+ and CD8+ lymphocytes starting from single-collection of mononuclear cells, stimulated with a viral-derived peptide mixture, in the presence of Il-4 and IL-7 (114). The multi-virus product was subsequently tested clinically in a miscellaneous group of HSCT recipients and produced a 94% response rate, with acceptable toxicity. The same group subsequently reported the possibility of producing HHV6-specific cell products (115). The final development was the inclusion of HHV6 specificity in an “off-the-shelf”, multi-virus T-lymphocytes program: this strategy has the advantage of storing ready to use third party T-lymphocytes products, overcoming the time limitation of *ad hoc* prepared cell concentrates. In a phase II study, a 67% response rate was observed in a limited number of HHV6 reactivation (62).

### BK Virus

BK viremia and viruria are common in HSCT recipients and are routinely monitored; BK positivity is frequently found also from the feces, suggesting that gastrointestinal mucosa may be an additional site of virus latency (137). It was found that in the early post-transplant phase, BKV-specific CD4 recovery was more common in patients without BK viruria. Conversely, beyond the sixth month after transplant, virus specific CD4 recovery was more frequently detected among patients with BKV viruria. Specific CD8 recovery occurred later and less frequently than CD4 one, and were more common among patients with BKV viruria. Differences were disclosed between CD4 and CD8 specific lymphocytes, as a naïve or CM phenotype accounted for a considerable proportion of CD4, whereas detected CD8 showed a predominant TEMRA phenotype (138).

Due to the severity of hemorrhagic cystitis, the use of CTL can be considered as an important therapeutic option. Unfortunately, BKV specific lymphocytes are present in low concentration even in reactive normal subjects (139). BKV is therefore generally among the target of broad-spectrum antiviral T-lymphocytes, and HC is among the indications of this therapeutic strategy (65). Nonetheless, specific anti-BKV T-lymphocytes can be selected by cytokine capture system and have been sometimes successfully delivered (139). Preliminary data on a phase II study have been presented about delivering of BKV-specific T-lymphocytes for the treatment of HC. In spite of the rather positive results, the study suffers the limitations of excluding patients with active GVHD. Moreover, CTL had been previously expanded from suitable random donors in order to avoid the risks of time to manufacturing and of failing to retrieve a sufficient number of lymphocytes (140).

### JC Virus

The data about monitoring and immunotherapy of JC infection are rather limited. JC virus is among the viruses whose DNA monitoring, both in urine and in peripheral blood is recommended (141). Investigating the appearance of anti-JC CD4+ and CD8+ lymphocytes through ELISPOT is feasible, with an underlying AML diagnosis and recipient age being reported as risk factors negatively linked to the establishment of an antiviral status (141). Donor derived VST can be produced after stimulation with viral proteins, and have been sometimes successfully delivered for the treatment of refractory PML (142). Data in HSCT setting, however, are missing.

### Conclusion

The almost totality of the studies about the management of viral infections after HSCT deal with DNA viruses, and most of them are addressed to CMV and EBV. Monitoring of virus specific immunologic recovery and adoptive immunotherapy is the best explored issues. On both fields, tetramer selection and magnetic immunoselection have proved to be the most promising approaches, each with specific pros and cons and without clear-cut evidence favoring one of the two. Monitoring of specific immune recovery allows an optimization of the therapeutic approach.

As for adoptive immunotherapy, the use of donor-derived lymphocytes is likely the optimal approach. However, in the setting of unrelated donor, it can be either troublesome if an additional leukapheresis is required in a “on demand” approach, or resource-wasting if lymphocyte collection is planned at the time of HSC harvesting. Moreover, donor lymphocytes may be unavailable. In any case, the outcome after delivering third party lymphocytes can be regarded as positive. Irrespective of the source, time to manufacturing is not a limitation in CMV and EBV, where large amounts of VST are easily retrieved, thus favoring an approach based on clinical needs.

The main matter of concern is the complex relationship between adoptive immunotherapy and GVHD. GVHD, either *de novo* or as flare up, is the most feared consequence of lymphocyte therapy; on the other hand, tapering of immunosuppression is a common measure in an attempt to control virus reactivation, further increasing GVHD risk in case of subsequent adoptive immunotherapy. Conversely, patients with active GVHD, notably on corticosteroids therapy, are generally excluded in clinical trials, thus ruling out the most troublesome patients and artificially reducing the impact of GVHD on the outcome of cell-therapy and viral diseases. Large scale availability of VST resistant to immunosuppressive agents is an expected development in order to allow clinical trials to be conducted also in patients with GVHD.

Lack of spontaneously retrievable virus-specific lymphocytes in healthy subjects, the possible failure to expand even available lymphocytes and the time required to achieve a suitable cell product, build up major limitations to immunotherapy in DNA virus infection other than CMV and EBV, even if a third party donor is selected. In these cases, an “off the shelf” and a pre-emptive clinical approach are favored. The availability of VST resistant to immunosuppressive agents is a need shared with CMV/EBV infection. A possible remedy to the failure of expanding VST for viruses other than CMV/EBV might be the engineering of TCR, as outlined in the ADV paragraph. Concern can be raised as to immunologic escape and vector-related events.

In spite of the evidence favoring adoptive immunotherapeutic in refractory viral infections, the available data seem to outline an approach based on local policies rather than on widely accepted strategies. Lack of clinical trials in patients with GVHD, that marks a difference with real life practice, may be a partial explanation.

Unfortunately, little is available on the issue of improving virus-specific immune recovery. The attempt at developing active immunotherapy strategies has led to questionable results at best. Relying on these data, this approach can be hardly regarded as promising.

In spite of its potential benefit and possible future developments, adoptive immunotherapy remains still a resource-wasting, cumbersome strategy, not devoid of toxicity. Its popularity seems to cover the lack of reliable alternatives. The availability of more effective anti-viral agents is probably the main unmet requirement.

## Virus as Immune Reconstitution Biomarkers

There has been historically great interest in finding good markers of immunologic recovery post HSCT with the final objective to personalize and optimize patient management. Based on the reported experience in solid organ transplantation (SOT), Torque Teno Virus (TTV), a single stranded DNA virus of the Anellovirus family (143, 144), has been studied as a possible non-pathogenic marker of immunocompetence. It can be retrieved from multiple biologic fluids in up to 100% of healthy subjects and is now generally considered as a component of the human virome (145), replicating in many organs and tissues, including T-lymphocytes; at the same time, TTV viremia is controlled by the presence of normally functioning T-lymphocytes (146). The characteristics of TTV are rather unique and may offer a novel instrument enabling to “measure” the immune function, beyond the limits of simple cell counts. In SOT, immunosuppressive drugs were associated to higher levels of TTV viremia whereas graft rejection is heralded by a decrease in DNA copies (147, 188). Confounding findings may be the less pronounced increase in TTV viremia in patients receiving sirolimus, possibly attributable to some anti-viral activity of m-TOR inhibitors (149), and the decrease in TTV DNA in patients receiving ATG, due to transient lack of substrate for viral replication (150). The data on SOT enabled other groups to investigate TTV as a marker of immune function in HSCT recipients, where the matter is made more intriguing by the complexity of the immune reconstitution process. Albert et al., retrospectively, and Wohlfarth et al., prospectively, observed a decrease in TTV DNA after delivery of conditioning regimen as a marker of lymphopenia, and its progressive increase along with lymphocyte recovery (151, 152). In both studies, the limited size of the series and the short period of observation (12 months) did not allow a correlation with the main clinical HSCT endpoints to be thoroughly looked for. More recently, a relationship has been suggested between the failure to clear TTV and major HSCT complications such as CMV reactivation (153) and GVHD (154). TTV viremia kinetic was then determined on a large series of HSCT recipients and analyzed in multivariable analysis: failure to clear TTV was linked to CMV, GVHD and unrelated donor, with patients bearing higher day 100 TTV levels showing a worse survival and a higher risk of severe aGVHD. The relationship between T-cell depletion and TTV is far from being disclosed, and available preliminary data open the question of whether a more accurate assessment of immunocompetence could be possible by TTV rather than by mere lymphocyte (155).

The ever-widening spectrum of treatments encompassed under the heading of HSCT makes the task far more troublesome than in the case of SOT.

## Effects of Viruses on Immunologic Recovery and Hsct Outcome

On the other hand, viruses remain one of the most acknowledged factors influencing or even remodeling immune recovery after HSCT, interfering with its outcome. Most of the available data in this field comes from CMV.

### CMV and Post-Transplant Immune Reconstitution

Historically CMV reactivation was associated with proliferative impairment in T lymphocytes of HSCT recipients (156), but more recent papers showed that these patients had faster CD8+ recovery (157). The simple CMV serological status seems to influence immune recovery, with D−/R+ status causing an increase in IFN-*γ* producing CD8+ lymphocytes and a reduction in multifunctional ones (39). Higher day 100 total CD8 counts were seen both in CMV seropositive and in CMV reactivators, especially after BM transplants (158). Itzykson et al. described that the mere CMV seropositivity, irrespective of CMV reactivation, resulted in a predominant recovery pattern, characterized by early CD8+ and late B-lymphocyte recovery; effector memory and late effector memory cells accounted for the majority of CD8+ lymphocytes, with the pattern being linked to a higher NRM (159). The burst of effector memory CD8+ after CMV reactivation had been observed up to one year in a pediatric series, with later central memory and naïve cell recovery after two years (160). In adults the expansion of effector memory CD8+ led to limited width repertoire and contraction of naïve T-cells, both CD4 and CD8, even in long term after transplant, with the hypothesis that the expansion of anti CMV-specific CD8 depresses the normal reactivity of the involved compartment (161). This difference seems to be attributable to immune aging, since very old CMV positive non-transplanted subjects exhibit a similar pattern of shrinkage of the T-cell repertoire (162).

Early expansion of NK after different kinds of HSCT has been diffusely described (163). Viral reactivation/infections seem to drive early (day 30) NK proliferation in the setting of T-repleted HSCT (164). CMV has been linked to distinct features of NK response. After CMV reactivation, NK preferentially express the activating NKG2C receptor instead of the inhibitory NKG2A, and the inhibitory “killer immunoglobulin-like receptor” (KIR) (165). The opposite does not work since NKG2C+ NK cell count does not predict CMV reactivation, at least in adults (166). NKG2C+ NK cells show a six-fold lower affinity with HLA-E bound proteins than NKG2A+ ones (167). The HLA-E bound CMV UL40 peptide has been shown to drive selectively the NKG2C+ NK clonal-like expansion and differentiation (168). KIR instead interacts with epitopes of conventional first-class HLA antigens, notably HLA-C. Among KIRs, KIR 2DL2/3 (CD158b) reacting with HLA-C1 is significantly more frequently expressed on NK in CMV positive recipients (159). After the challenge with CMV, NK modify their CD56 positivity from bright to dim and acquire CD57 positivity as a marker of a “memory” adaptive phenotype. CMV-shaped NK react releasing a burst of gamma-interferon to further challenge through HLA-E bound peptides, with the aim of protection from further CMV reactivation (169). Thus, in these patients, a unique KIR expressing CD56+CD57+NKGC2+ CD8+ T-cell subpopulation may be an additional marker of CMV-driven immune recovery after HSCT (170). In a study on cord blood recipients, CD56 (dim)KIR+ NKG2A-cells were the expanding NK population, without detectable NKG2C cells. Possible relevant differences are hence disclosed between an adult donor and a naïve immune system (163). The difference between adult and cord blood HSCT is underscored by an additional study failing to disclose a rapid adaptive CMV-induced NK cells in UCB recipients, late-occurring only in patients with high viral load (169). In experimental animal models, CMV proved to be the driver of the adapted NK response and adapted NK proved to be effective in clearing CMV at virus re-challenge, as could be hypothesized from clinical studies (171). The wide heterogeneity of HSCT settings and some contradictory results (46) raise the question as to whether CMV-linked features of NK response are invariably to be expected after every type of HSCT. For example, on a series of MUD HSCT recipients, a significant increase of NK bearing an adapted phenotype was limited to cases where BM was the stem cell source (158). Bigger and more homogeneous studies need to be carried out to elucidate better these interactions.

Further hallmarks of CMV reactivation are large granular lymphocytes (LGL) and *γδ* T-cell expansion. LGL increase is a late phenomenon, peaking beyond one year after HSCT; the expanded population bears a CD8+ phenotype and poses questions as to its biological significance, since monoclonal TCR rearrangement can be observed in a substantial proportion of cases. In spite of long-term persistence of restricted LGL in a minority of cases, the available data point out to a reactive rather neoplastic nature (172). *γδ* T-cell expansion in characteristically confined to the V*δ*-2- compartment, with possible effects on the susceptibility to infectious complications (173). Differently from normal adults, T-*γδ* proliferation in HSCT recipients presents an adaptive pattern primarily shaped by CMV, including the proliferative potential after re-challenge (174).

### CMV and Transplant Outcome/GVHD

It is of much interest to use all these data regarding anti-CMV immune response to understand possible interfering mechanisms with immunological aspects of HSCT. Apart from the direct infectious risk, in fact, the relationships between CMV reactivation and outcome of HSCT are not univocal and are still debated. CMV exerts a deep influence on immune recovery, bearing the potential of interfering with the main determinants of HSCT outcome, as engraftment, GVHD, NRM and relapse of the underlying disease. One example is that D−/R+ matching was postulated to lead to the proliferation of recipient CD8, which can jeopardize donor immune recovery and the achievement of full donor chimerism (175, 176). An increase in NRM has been commonly reported as a consequence of CMV on HSCT outcome (177–179) and of CMV-driven immune recovery profile (159), of course, at least in part, due to the morbidity and mortality linked to the CMV infection per se.

The question about GVHD is more intriguing and less elucidated. GVHD is an obvious risk factor for CMV reactivation, but whether CMV accounts for an increased risk of GVHD is more controversial. Bidirectional relationship between acute GVHD and CMV have been extensively described (179, 180) but the underlying causative factors remained speculative. The cross-reactivity between CMV-specific T-cells and host allo-antigens is the most appealing link between CMV and GVHD (181, 182), but the question in still open (31). The CMV-related imbalance in T-reg recovery has been claimed to explain the increased GvHD (183). Virus-related overexpression of mismatched class I, mainly HLA-C (170), and class II, mainly HLA-DPB1 (181), may play a role in settings other than HLA-identical HSCT, considering also HLA-DPB1 is not considered in defining a 10/10 matched unrelated donor. As for non-conventional class I HLA antigens, it has not been thoroughly investigated. No data are available about a possible under-expression of the immune-regulatory HLA-G by CMV, as documented after HSV reactivation (184). Some indirect evidence can be instead drawn from HLA-E expression. HLA-E shows a low degree of polymorphism, with the majority of the human population having either HLA-E*01:03 or HLA-E*01:01, with HLA-E*01:01 being linked to an increased risk of both GVHD and disease relapse. As previously mentioned, HLA-E bound peptides interact with NK through the inhibiting NKG2A and the activating NKG2C receptor, but HLA-E is also recognized by T-cell (167). The complex network connecting CMV, HLA-E expression, peptide binding and NK activation (167, 182) may elicit a T-lymphocyte reaction, notably in a context of HLA-E*01:01mismatch.

Along with the appearance of papers underscoring the negative effect of CMV reactivation/infection on the outcome of HSCT, a parallel literature points out to its possible beneficial effects. As the detrimental activity was mainly related to an increase in NRM, the potential benefits included a lower risk of relapse and a better disease-free survival (DFS). In 2006, a significant survival benefit had been demonstrated for HSCT recipients experiencing CMV reactivation but not disease (185) Explanations to this observation were speculated to be through protection from relapse by CMV-induced pseudo-clonal proliferation and “memory”-like adaptation of NK cells (165) and through an increased graft *versus* leukemia (GVL) effect through CMV-conditioned NK cells (170). Most of the research has been progressively focused to the target CMV and DFS in AML patients. Positive CMV antigenemia significantly reduced the risk of relapse in AML patients but the advantage in DFS was counterbalanced by an excess in NRM, confirmed also in an EBMT survey (177–179). In multivariable analysis, CMV-reactivation proved to be among the independent variables predicting a better DFS in AML patients, with simultaneous CMV-reactivation and chronic GVHD being associated to a highly significant advantage in terms of survival (186). Considering only transplants in first CR, the DFS advantage overrode the worse NRM, thus leading to a balance significantly favoring CMV reactivators (187), with greater net benefit to be expected in patients receiving T-cell repleted grafts (188). Some more insights can be derived from haploidentical HSCT, where the rate of CMV reactivation is notably high. In this setting, the presence of one or more class I MHC genotypes, characterized by a higher efficiency in presenting CMV antigens, had been linked to a lower relapse and non-relapse mortality rate without an excess of GVHD (189), also in multivariable analysis (31).

To sum up, the above data suggest that CMV reactivation may somewhat reinforce a GVL effect in the setting of HSCT, especially in AML patients. The benefit, if any, has to be attributed to its remodeling effect on immune recovery. In order to offer a satisfactory explanation of this phenomenon, the main attention has been driven by the NK activation and by the *γδ* lymphocytes expansion, although the available evidence is far from having thoroughly clarified the issue. CMV-induced adapted CD56(dim)CD57+NKG2A-NKG2C+KIR+ NK population may react against proteins bound to HLA-E bearing leukemic blasts; the switch from the more selective inhibitory NKG2A to the activating NKG2C in CD57+ NK may exert a key role (190). On the more restricted field of mismatched and haploidentical HSCT, the missing self-antigen, perceived by the inhibitory KIRs, as HLA-C sensing KIR 2DL2/3, could trigger an adapted NK reaction against the HLA mismatched leukemic cells (191). As said before, *γδ* T-lymphocytes proliferating compartment is invariably the V*δ*-2- one. This increase in V*δ*-2- *γδ* lymphocytes resulted as a favorable predictor of post-HSCT DFS in acute leukemia (192). Lastly, cross-reactivity was preliminarily disclosed between CMV and leukemic cells (193), with need for further evidence supporting this.

### Other Viruses and Transplant Outcome

In spite of the outmost interest raised by PTLD and adoptive immunotherapy, the data about EBV reactivation and post-HSCT immune recovery are rather scant. Viral load has been reported to impair generically both B- and T-cell recovery (194, 195). The expansion of CD8+ effector memory cells, characteristically related to CMV, has not been observed after EBV and ADV reactivation (196). A proliferation of *γδ* T-lymphocytes, bearing analogy to CMV reactivation, has been described (197), but other investigators were not able to confirm these data (198). An increase in double negative T-lymphocytes has been linked to EBV re-activation (199). Surprisingly, and somewhat similarly to CMV, EBV reactivation without PTLD was linked to earlier NK recovery and better survival, mainly attributable to a lower relapse rate, irrespective of the type of transplant (200). These data are however more explained by Minculescu et al. findings, linking generically early NK proliferation to viral infection and higher NK counts to lower TRM (164). The lines of immunologic evidence linking CMV reactivation to AML control are lacking in the setting of EBV. In a study linking acute and chronic GVHD to CMV reactivation, the authors failed to demonstrate any relationship between EBV reactivation and GVHD (196).

Even fewer is known about the specific effects of other viruses. An old observation linked HHV-6 reactivation to persistent post-HSCT lymphopenia (201). The same authors linked an impaired anti-CMV immune response to HHV-6 reactivation (202). In line with these data, Quintela et al. described delayed T-cell recovery and increased risk of CMV infection in patients reactivating HHV-6 (133). Higher HHV6 viremia, in a pediatric series, seemed to hamper long term T-cell reconstitution, both CD4 and CD8, whereas the effector memory compartment resulted unaffected, suggesting less impairment of short term immune recovery (203). Clinically, HHV6 reactivation occurs in about 50% of HSCT recipients, with higher risk of acute GVHD and NRM and worse OS, both in pediatric and in adult series (204–207). These effects seem stronger in MAC transplant (207–209) and in UCB (133). Due to the lack of specific researches, the possible relationships between HHV6, acute GVHD and HSCT outcome, are merely speculative (210).

A relationship was found between Herpes simplex early activation, presence of the POL herpetic antigens in the skin and GVHD; according to the authors, virus-induced activation of dendritic cells could have exerted a key role (211). Moreover, Herpes simplex causes a lower expression of HLA-G on endometrial decidual cell; HLA-G immune-inhibitory effect may play a role in maternal-fetal tolerance during pregnancy; speculatively at least, these data may account for another link between Herpes simplex and GVHD (184).

In spite of its relevance in pediatric HSCT, little is known about the relationships between adenovirus and immune recovery, apart from a generic report of delayed T- and B- cell reconstitution (212). Little is known also about the effects of ADV infection and HSCT outcome, apart from the mortality of the viral disease *per se*. A single study pointed out to ADV stool positivity as a risk factor for intestinal acute GVHD (213). More generally, DNA virus infections have been associated to worse HSCT outcomes (214), with only scant evidence suggesting a protective role against AML relapse after viral infections other CMV (187).

### Conclusion

Even more than in the management of virus infection, precise and definite data on the interference of viruses with immune recovery after HSCT are lacking and are mainly restricted to the CMV issue. The reported investigations point out to the potential of CMV, and marginally of other DNA viruses, of shaping immune recovery, although the results are not univocal and do not encompass the wide spectrum of transplant-related variables and complexity.

There is some concordance as to the fact that CMV may increase GVHD risk and possibly the risk of other infectious complication, thus worsening NRM and OS. Apart from possible homologies between viral and human sequences, the evolution from sibling to MUD, to HLA mismatched HSCT, calls progressively for a study of the role of non-conventional and conventional MHC mismatches as a link between CMV immune response and GVHD.

According to other report, CMV could decrease the risk of baseline disease relapse, at least in the setting of AML, possibly also involving some of the pathways linking CMV to GVHD. It is debatable whether this eventually translates into an advantage or a reduction in survival. Underlying diagnosis, donor selection, conditioning regimen and type of transplant give rise to multiple combinations with different GVHD, relapse and infectious risks, thus offering some explanation for contradictory results.

Investigations on the effects of other viruses on immunologic recovery and HSCT outcome are warranted and can be expected to disclose relevant issues in the management of HSCT patients.

## HSCT as Immunotherapy in Viral Infections

Despite viruses being one of the main complications of HSCT, HSCT was used to try and cure some viral illnesses through exerting an immunotherapeutic effect, at least in some selected viral infections.

### HIV

In 2009, the case was reported of a HIV patient undergoing HSCT from a CCR5 Delta32/Delta32 donor, who achieved long term HIV-free survival, lasting over anti-retroviral therapy (ART) discontinuation (215). The persistence of remission was confirmed at longer follow-ups of the same patient, commonly known nowadays as the Berlin patient (216). Moreover, HIV was not found anymore in any biological samples of the patients and the antibody response weaned over time which was interpreted as an additional proof of recovery (217). A possible explanation for the Berlin patient outcome could be the natural HIV refractoriness of the donor. Although CCR5 Delta32/Delta32 is present in about 1% of the general population (70), only one more documented case of HSCT from a CCR5 Delta32 homozygous donor, has been reported in a HIV patient; HIV viremia became undetectable but the patient died of his underlying lymphoma, thus hindering any evaluation of long-term post-transplant outcome of the HIV infection.

Beyond the above considerations pointing out to HSCT cure as a fortuitous event in HIV, some evidence seems to show an intermediate outcome in other HIV patients after HSCT. A deep, progressive reduction in HIV reservoir was observed in a small series of long-term survivors after HSCT from wild-type CCR5 donors (218). Similar results were reported in other small series or case reports, with anti-HIV response lasting over ART discontinuation and sometimes ending into an acute viral rebound phase, suggesting some kind of immunologic escape (219, 220). As a whole, these data seem to account for a graft *vs*-HIV effect (208) and enabled to look for immunologic strategies to improve post-HSCT HIV control (70). Patel et al. raised and expanded *in vitro* HIV-specific T lymphocytes from HIV-naïve healthy donors. Anti-HIV T-lymphocytes reacted *in vitro* against different viral epitopes. Interestingly, most of the CD8 lymphocytes exhibited a CD45RA− CD62L− effector memory phenotype, similar to what happens after challenging with other viruses, such as CMV. A proportion of CD45RA− CD62L+ central memory cells was also obtained, possibly more capable of long term disease control (70). More recently, the same group reported the possibility of producing GMP-compliant HIV-specific T-lymphocytes with wide viral epitope recognition and high *in vitro* activity. Again, effector memory cells accounted for the majority of CD8 lymphocytes, still with a minor proportion of central memory phenotypes (221).

### HTLV-1

HSCT has been proposed and currently performed as possible curative approach to another retrovirus, HTLV-1, etiologic agent of adult T-cell leukemia/lymphoma (ATLL) (222). According to non-recent data, the retrovirus HTLV-1 infects about twenty million people worldwide, mostly in East Asia (223), with <5% of them eventually developing ATLL (224). The viral trans-activator TAX plays a central role in the virus-related oncogenesis by exerting multiple deregulatory activities on key genes involved in T-cell homeostasis (222). The clear-cut relationship between virus and neoplasia and the deriving possibility of controlling ATLL through an effective GVL targeting virus specific antigens confer unique features to HSCT in this indication. HTLV-1 bZIP factor has been proposed as a possible target of immunotherapy. Patients affected by ATLL and asymptomatic HTLV-1 carriers exhibit tolerance towards this viral antigen as testified by the lack of reactive T-lymphocytes. Conversely, donor derived immune system after HSCT does express bZIP specific CD4+ T-cells, thus bearing the potential of targeting virus infected cells (225). The matter concerning TAX is more trivial, since TAX is not always expressed in ATLL cells, and sometimes the appearance of TAX expression seems to herald some kind of immune escape and an overwhelming relapse (224). Conversely, donor derived TAX-specific viral specific T-cells are found in the setting of HSCT (226), and have proved to be effective and long lasting in inhibiting HTLV-1 infected cells both *in-vivo* and *in vitro* (227). The experience with HSCT has given rise to alternative strategies, in order to overcome HSCT itself and the related risks. In particular, creating autologous TAX-specific viral specific T-cells, *via* a TAX-directed “vaccine” has been hypothesized (226–228).

### Other Viruses

Lastly, the issue of HSCT as an immunotherapy in T/NK EBV-related lymphoproliferative disorders deserves some remarks. In immunocompetent subjects, EBV proliferation in T/NK cells may cause a spectrum of diseases ranging from chronic active EBV infection (CAEBV) to extra-nodal T/NK lymphoma and NK leukemia (229). CAEBV mainly affects children, and NK/T-cell leukemia/lymphoma mainly young adults (230, 231). Case reports and small series propose HSCT as an effective treatment option in both conditions (229). In this setting, evidence has been reported of an immunotherapeutic effect exerted by HSCT (229, 232) and even of the possibility of improving the outcome of HSCT through the infusion of donor-derived targeted viral specific T-cells (233).

### Conclusion

The role of HSCT to treat virus-related disease is doomed at being a marginal one and is mentioned here for the sake of completeness. Severity of the disease, lack of effective alternative treatments, and availability of a viral molecular marker exerting a key pathogenetic role targeted by HSCT are main requirements to be simultaneously satisfied in order to undergo the otherwise unacceptable risks of HSCT. Therefore, extension of the indications beyond HTLV-1 and EBV-related diseases is unlikely. HIV seems to be a theoretical rather than practical indication, even if HSCT proved to be feasible in HIV patients affected by neoplastic diseases.

## Author Contributions

CA designed the manuscript structure, organized the literature search, and wrote the first draft. GS, GG, FG, MG, FC, FS, LB, and FO contributed to the literature search/revision and to the manuscript writing. GS organized the references editing. FO made the final revision, manuscript editing, and approval. All authors contributed to the article and approved the submitted version.

## Conflict of Interest

The authors declare that the research was conducted in the absence of any commercial or financial relationships that could be construed as a potential conflict of interest.
